# Learning from the past? How biased memories of the pandemic endanger preparation for future crises

**DOI:** 10.1002/ctm2.1510

**Published:** 2023-12-07

**Authors:** Philipp Sprengholz, Luca Henkel, Robert Böhm, Cornelia Betsch

**Affiliations:** ^1^ Department of Psychology University of Bamberg Bamberg Germany; ^2^ Institute for Planetary Health Behaviour University of Erfurt Erfurt Germany; ^3^ Department of Implementation Science Bernhard Nocht Institute for Tropical Medicine Hamburg Germany; ^4^ Kenneth C. Griffin Department of Economics University of Chicago Chicago Illinois USA; ^5^ Department of Economics University of CEMA Buenos Aires Argentina; ^6^ Faculty of Psychology University of Vienna Vienna Austria; ^7^ Department of Psychology University of Copenhagen Copenhagen Denmark; ^8^ Copenhagen Center for Social Data Science University of Copenhagen Copenhagen Denmark

**Keywords:** pandemic preparedness, recall bias

As the world transitions to a postpandemic phase, societies are looking to evaluate their past responses to COVID‐19 and prepare for future crises. However, a recently published series of studies^1^ sheds light on a concerning issue; sustained societal polarisation between the vaccinated and unvaccinated is distorting the accuracy of people's recall of the pandemic, fuelling societal conflict and complicating preparation for future pandemics. Here, we summarise the key findings and elaborate on the implications for clinical practice.

## 1 DISTORTED RECALL AND ITS ORIGINS

According to psychological research, an individual's retrospective recollection and interpretation of historical events can be distorted by their current motives or worldviews.^2^, ^3^, ^4^ In light of the significant observed polarisation between the vaccinated and unvaccinated during the pandemic,^5^ we sought to investigate the extent to which these two groups overestimated or underestimated their own past risk perceptions, behaviours and trust in government and science, and how these distortions affected their evaluation of past political actions. In a first study conducted in Germany, participants were asked about their pandemic‐related perceptions at two different time points: in the first year of the pandemic (2020) and again at the end of 2022. At the second time point, they were also asked to recall their earlier responses, enabling us to compare actual and recalled answers. Strikingly, many respondents’ recollections were distorted, deviating systematically from their previous responses in different directions, depending on whether they were vaccinated or unvaccinated. For instance, while vaccinated individuals tended to *over*estimate their past risk perceptions and trust in science, unvaccinated participants tended to *under*estimate them. This distortion was especially pronounced among individuals who identified strongly with their vaccination status – that is, those for whom being vaccinated or unvaccinated was an important element of their social identity. Distortion was not a result of mere forgetfulness, as the introduction of a significant monetary incentive for correct recall in a follow‐up study served to reduce recall bias. Importantly, distortion was significantly associated with people's evaluation of past political actions; greater recall bias came with a more extreme evaluation of political action – in either direction.

## 2 IMPLICATIONS FOR FUTURE CRISES

The systematically divergent narratives about the pandemic could potentially perpetuate societal polarisation and complicate preparation and action in future crises. If an individual (vaccinated or unvaccinated) misremembers their perceptions of risk during the pandemic, it may significantly influence their response to upcoming threats. For example, an unvaccinated individual who downplays past risks may be less inclined to follow health regulations and recommendations in the future. Indeed, another international study across 10 countries (including Germany, Mexico, Japan, the United Kingdom and the United States) confirmed that polarised perceptions of the past are associated with decreased intention to follow future pandemic regulations, with increased desire to punish politicians and scientists, and with increased distrust in political institutions. Those findings highlight the potentially negative societal consequences of polarised memories and evaluations of the past. For instance, while 84% of Mexican respondents expressed a willingness to follow recommendations and regulations in a similar future pandemic, only 64% of American respondents intended to do so. Those findings underscore the need to develop strategies to mitigate societal polarisation and indicate that decision makers must consider the longer‐term consequences for societal cohesion and trust when responding to such challenges.

## 3 CONSEQUENCES FOR CLINICAL PRACTICE

As frontline responders and influencers of public health behaviours, clinicians need to be aware of recall distortions and their potential effects on perceptions and actions in upcoming health crises. The fact that recall is motivationally biased towards the present may also affect the assessment of a patient's medical history if that history is self‐reported. How people recall their past illnesses may be systematically distorted, potentially leading to incorrect anamnesis. This could affect shared decision‐making processes, where personal values and subjective accounts play a major role.

Many of the participants in our studies identified strongly with their vaccination status. In this regard, clinicians could contribute to the reduction of societal polarisation by helping to (re)frame vaccination as a health decision rather than a matter of identity or group membership. Clearly, strong identification may complicate conversations about future vaccinations, especially in the case of critical patients. For that reason, clinicians may be well advised to refresh their communication skills – for instance, by accessing resources that explain common misperceptions and arguments used to discredit vaccination. As one scientifically sound approach, motivational interviewing advocates an empathetic approach to such conversations,^6^ offering evidence‐based information and corrections to address any underlying misinformation.^7^ The website at https://jitsuvax.info/ offers training for recognising typical arguments against vaccination and possible responses.

Our findings indicate that people's experiences of the COVID‐19 pandemic may make them less likely to follow future pandemic regulations and recommendations. Clinicians should therefore emphasise the importance of adhering to preventive measures in order to maintain overall health. Given the increased distrust in political institutions highlighted by the studies, clinicians should be mindful in that regard and work towards building trust in the healthcare system.

## AUTHOR CONTRIBUTIONS

Not Applicable.

## CONFLICT OF INTEREST STATEMENT

The authors declare no competing interests.

## FUNDING

The authors have nothing to report.

## ETHICS STATEMENT

Not Applicable.

## Data Availability

Not Applicable.
